# Prevalence and Factors Associated With Cancer‐Related Fatigue Among Children and Adolescents Undergoing Cancer Treatment: A Systematic Review and Meta‐Analysis

**DOI:** 10.1002/cam4.70502

**Published:** 2024-12-11

**Authors:** Sapna Oberoi, Beili Huang, Rasheda Rabbani, Nicole Askin, George Okoli, Richa Jain, Lillian Sung, Maya M. Jeyaraman, Alyson Mahar, Roberta Woodgate, Ryan Zarychanski

**Affiliations:** ^1^ Department of Pediatric Hematology/Oncology Cancer Care Manitoba Winnipeg Manitoba Canada; ^2^ Department of Pediatrics and Child Health University of Manitoba Winnipeg Manitoba Canada; ^3^ Department of Community Health Sciences, Max Rady College of Medicine University of Manitoba Winnipeg Manitoba Canada; ^4^ Department of Obstetrics and Gynecology University of Calgary Calgary Alberta Canada; ^5^ George & Fay Yee Center for Healthcare Innovation University of Manitoba Winnipeg Manitoba Canada; ^6^ Neil John Maclean Health Sciences Library University of Manitoba Winnipeg Manitoba Canada; ^7^ Postgraduate Institute of Medical Education and Research Chandigarh India; ^8^ Division of Haematology/Oncology The Hospital of Sick Children Toronto Ontario Canada; ^9^ Division of Cancer Care and Epidemiology, Queen's Cancer Research Institute Queen's University Kingston Ontario Canada; ^10^ College of Nursing University of Manitoba Winnipeg Manitoba Canada; ^11^ Department of Internal Medicine, Rady Faculty of Health Sciences University of Manitoba Winnipeg Manitoba Canada

**Keywords:** adolescents, cancer, chemotherapy, children, fatigue, risk factors, treatment

## Abstract

**Background:**

The literature on fatigue in children and adolescents undergoing cancer treatment is highly variable, creating uncertainties about its prevalence and identifying those at higher risk.

**Objectives:**

The primary purpose was to describe the prevalence of fatigue among patients (< 21 years) undergoing cancer treatment across cancer types. Secondary outcomes included the prevalence of severe fatigue and factors associated with fatigue.

**Methods:**

Systematic searches of MEDLINE, Embase, Cochrane Central Register of Controlled Trials, CINAHL, and PsycINFO were conducted from inception to May 22, 2023. Two reviewers independently identified relevant citations and extracted data. Pooled prevalence estimates were derived using an inverse variance, random‐effects model. We used Joanna Briggs's critical appraisal checklist to assess study quality. (PROSPERO: CRD42020179307).

**Results:**

We included 47 studies: 26 for prevalence and 29 for factors associated with fatigue. The pooled prevalence of fatigue was 73% (95% [Confidence Interval, CI: 66%–79%; *I* ^2^ 96%; 26 studies; 2699 patients], and severe fatigue was 30% [95% CI 14%–46%, *I* ^2^ 98%; 8 studies; 1027 patients]). Subgroup analyses based on cancer type, study design, fatigue scale, fatigue reporting personnel, sample frame, and response rate did not reveal significant differences in fatigue prevalence. Fatigue prevalence significantly differed by treatment setting (inpatient [83%] vs. outpatient [55%] vs. inpatient and outpatient [69%]; p: 0.02). Due to considerable heterogeneity among studies, data on fatigue‐associated factors are presented descriptively.

**Conclusions:**

The prevalence of fatigue among children and adolescents undergoing cancer treatment is variable but notably high. Systematic evaluation of factors associated with fatigue is essential to understanding which children are at high risk of developing fatigue.

**Trial Registration:**

PROSPERO: CRD42020179307

## 1 Introduction

Fatigue is a debilitating symptom commonly experienced by children and adolescents with cancer throughout their cancer journey [1]. Fatigue can result from a range of factors, including cancer itself, treatment‐related toxicities such as pain, infection, and anorexia, as well as psychological distress, anxiety, depression, and sleep disturbances. Fatigue significantly impacts children's ability to engage in daily activities, such as attending school, participating in sports, and maintaining social relationships. Beyond physical tiredness, it also affects their cognitive and psychological functioning, leading to psychological‐emotional distress, particularly in adolescents with cancer [2, 3]. Fatigue has been reported to be one of the most crucial factors impacting the quality of life of these children and adolescents [4].

The reported prevalence of fatigue among children and adolescents with cancer during treatment is heterogeneous, varying from 14% to 67%, and has been shown to depend on various demographic and treatment‐related factors [5, 6, 7, 8]. While a few systematic reviews have analyzed interventions to mitigate fatigue in adults and pediatric patients with cancer, none have evaluated the prevalence and correlates of fatigue in the pediatric population during treatment [9, 10, 11]. Such a review can help assess the overall prevalence of fatigue in children with various cancer diagnoses during treatment by synthesizing data from multiple studies to provide a more precise estimate of fatigue prevalence. Investigating potential factors associated with fatigue is also essential to identify children and adolescents at risk of experiencing more fatigue during cancer treatment. Overall, this information can provide evidence‐based insights to inform the development of strategies for screening, preventing, and treating fatigue in children and adolescents during treatment.

Therefore, the primary objective of this systematic review and meta‐analysis was to determine the prevalence of fatigue, regardless of its severity and etiology, in children and adolescents receiving cancer treatment. The secondary objectives included assessing the prevalence of severe fatigue and identifying factors associated with the occurrence of fatigue of any severity in this patient population.

## 2 Methods

We conducted this systematic review following the methodological approaches outlined in the Cochrane Handbook for Systematic Reviews of Interventions and adhered to the Meta‐analysis of Observational Studies in Epidemiology (MOOSE) guidelines for reporting meta‐analyses of observational studies [12, 13]. This review was registered with PROSPERO (CRD42020179307) [14].

### 2.1 Search Strategy and Study Selection

A systematic search was conducted using Medline (Ovid), Embase (Ovid), Cochrane Central (Wiley), CINAHL (EBSCO), and PsycINFO (Ovid) from inception to May 22, 2023 (Table ). Reference lists of narrative and systematic reviews and the included studies were screened for additional citations. Additionally, we searched the gray literature to capture relevant studies.

### 2.2 Inclusion and Exclusion Criteria

Studies were included if they met the following criteria: (1) Participants were under 21 years of age with cancer or hematopoietic stem cell transplant (HCT) recipients; (2) the intervention included chemotherapy, radiotherapy, surgery, HCT, targeted therapy, or immunotherapy for cancer treatment; (3) reported prevalence, risk factors, or other variables associated with fatigue irrespective of the cause of fatigue; and (4) the study design was randomized controlled trials (RCTs), cohort studies, controlled before‐and‐after studies, or cross‐sectional studies. RCTs and controlled before‐and‐after studies had to report the prevalence of fatigue at baseline before the initiation of the intervention, provided they did not have the presence or severity of fatigue as an eligibility criterion for enrolling patients. Studies were excluded if they (1) included < 75% of children and adolescents with cancer; (2) exclusively included children and adolescents with relapsed/refractory cancer or those receiving palliative care or had > 25% of children and adolescents with relapsed/refractory cancer or those receiving palliative care; (3) solely included childhood cancer survivors or had > 25% of the population off cancer treatment; (4) did not report any outcomes of interest to this review; (5) reported fatigue only at cancer diagnosis; or (6) were non‐English language studies.

### 2.3 Outcomes

The primary outcome of interest was the prevalence of overall self‐ or proxy‐report fatigue measured by any fatigue assessment scale, except when fatigue was reported as toxicity using Common Terminology Criteria for Adverse Events (CTCAE) grading. Secondary outcomes included the prevalence of self‐ or proxy‐report study‐defined severe fatigue measured by any fatigue assessment scale, except for CTCAE, and study‐reported patient‐, disease‐, or treatment‐related factors associated with the presence of fatigue.

### 2.4 Data Extraction

Two reviewers (SO, RJ, BH, GO, or OL) independently evaluated the titles and abstracts identified by the search strategy. Any citation deemed potentially relevant by either reviewer was retrieved in full‐text format and assessed for eligibility. Discrepancies between the two reviewers were resolved through consensus, with adjudication by a third reviewer (GO or SO) if necessary. We extracted the following data: (a) Study‐level variables, including study design and setting (inpatient vs. outpatient), year of enrollment and publication, country of study, inclusion and exclusion criteria, and quality assessment items; (b) Population‐, disease‐, and treatment‐related variables: such as age at diagnosis, sex and ethnicity of participants, cancer type, cancer stage (nonmetastatic vs. metastatic), percentage of participants with relapsed/refractory disease, type of cancer treatment, and phase of cancer treatment; (c) Outcome‐related variables including the type of fatigue assessment scale used, outcome definition (i.e., specific diagnostic criteria or fatigue assessment scale cut‐off), time point of fatigue assessment, the person reporting fatigue, and the number of patients assessed for or presenting with fatigue and severe fatigue and factors associated with fatigue. In case of missing or unclear data, authors of the individual studies were contacted to provide the required information. Studies using the same patients or datasets were grouped as companion studies and treated as a single study in this systematic review. When studies reported fatigue prevalence from both patient and parent perspectives, the patient‐reported data were used in our meta‐analysis. We used the average prevalence for longitudinal studies that provided prevalence estimates at multiple time points during cancer treatment.

### 2.5 Study Quality Assessment

Two reviewers (SO and RJ, BH, OL, or GO) assessed the quality of observational studies using the Joanna Briggs Institute's (JBI) critical appraisal checklist for studies reporting prevalence data [15]. Nine domains of each study were considered for quality assessment, including sample frame, sampling method, sample size, study setting, sample coverage, validation of fatigue scale, fatigue measurement, statistical analysis, and response rate (Table S2).

### 2.6 Data Synthesis

We performed descriptive statistics using Microsoft Excel 2019 [Excel v15, Microsoft Corp., Redmond, WA, United States of America (USA)] and pooled aggregate data at the study level. If study‐level proportions and standard errors were not reported, they were calculated before meta‐analyses. We averaged the prevalence estimates for longitudinal studies reporting prevalence estimates at different time points during cancer treatment. The weighted summary proportions of fatigue and corresponding 95% confidence intervals (CI) were calculated by pooling the study‐specific estimates using generic inverse variance random‐effects models. Statistical heterogeneity between studies was assessed using the *I* ^2^ statistic, which describes the percentage of total variation across studies due to heterogeneity rather than chance; values around 30%–60% represent moderate heterogeneity, 50%–90% substantial heterogeneity, and 75%–100% considerable heterogeneity [16]. Potential publication bias was evaluated using visual inspection of funnel plot analysis and Egger's regression test for the primary outcome of fatigue prevalence [17, 18]. To explore sources of heterogeneity and to provide estimates of fatigue prevalence among relevant subgroups, stratified/subgroup analyses were planned based on a priori‐defined subgroups based on age, sex/gender, presence of mood or anxiety disorders and sleep disorders, cancer type and stage, treatment type, treatment setting, the person reporting fatigue, type of fatigue scale used, study type, and adequate sample frame, sampling and response rate [14]. These subgroup comparisons provided detailed insights into how fatigue varied within distinct patient populations and treatment contexts. We used the *p* value for subgroup difference to assess whether the prevalence of fatigue varied across subgroups, with a *p* value < 0.05 indicating a statistically significant effect. The meta‐analysis was performed using the metafor R package in R software (version 4.3.0) [19].

## 3 Results

**FIGURE 1:**
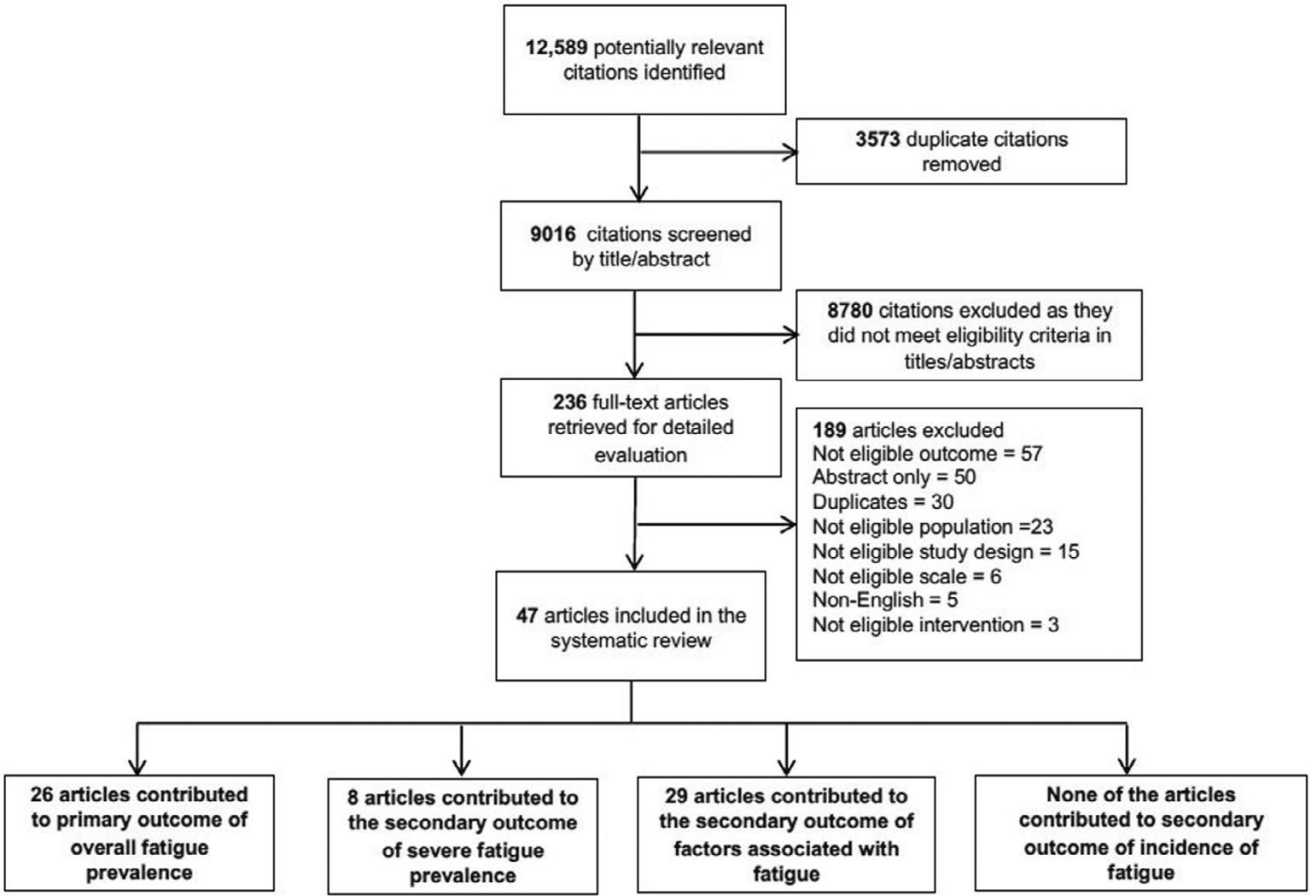
PRISMA flowchart of the selection of the included articles.

We identified 12,589 citations and screened 9016 after removing duplicates. Following a full‐text review of 236 articles, 47 studies met the eligibility criteria and were included in the review (Figure 1).

### 3.1 Study Characteristics

Table 1 lists the baseline characteristics of all included studies. Of the 47 studies [5, 6, 8, 20, 21, 22, 23, 24, 25, 26, 27, 28, 29, 30, 31, 32, 33, 34, 35, 36, 37, 38, 39, 40, 41, 42, 43, 44, 45, 46, 47, 48, 49, 50, 51, 52, 53, 54, 55, 56, 57, 58, 59, 60, 61, 62, 63, 64] 26 contributed to the primary outcome of overall fatigue prevalence [5, 6, 8, 20, 21, 22, 23, 25, 26, 27, 30, 33, 34, 35, 36, 37, 40, 42, 44, 45, 49, 50, 53, 54, 56, 62] 29 to factors associated with fatigue [5, 20, 21, 22, 24, 26, 28, 29, 32, 35, 36, 38, 39, 41, 42, 43, 44, 46, 47, 51, 52, 55, 56, 57, 58, 60, 61, 65, 66] and 8 to the prevalence of severe fatigue [8, 20, 40, 49, 53, 54, 62, 67]. Geographically, 47% of studies were conducted in the USA, 13% in the USA and Canada, 6% in China, 4% in Canada, and 4% in Turkey. Prospective cohort studies comprised 60%, while cross‐sectional and phase 3 RCTs comprised 30% and 9%, respectively. Among the 30 studies from the USA and Canada, 25 reported enrolments of non‐white participants with a median representation of 28% (range: 8%–75%). Sex/gender distribution was reported in all studies, with a median of 56% males (range: 41%–80%). Cancer diagnoses varied widely: 64% of studies included patients with different cancer types, 21% focused solely on acute lymphoblastic leukemia (ALL), and 4% included both acute myeloid leukemia (AML) and ALL. Two studies exclusively enrolled patients with Central Nervous System (CNS) tumors. Regarding the study setting, 47% included patients receiving inpatient and outpatient treatments, while 19% and 17% focused exclusively on inpatient or outpatient settings. Modalities of cancer treatment included chemotherapy (51%), chemotherapy/radiation/surgery (13%), chemotherapy/radiation (4%), radiation (2%), and HCT (2%).

**TABLE 1:** Summary of studies included in the systematic review (*N* = 47).

Study ID (author and year)	Country of study	Study type	Study setting	Inclusion of HSCT patients	Age of participants (years), mean (range)	Ethnicity (non‐White%)	Sex (% male)	Type of cancer	Total no. of participants in study	Person reporting fatigue	Fatigue prevalence reported
Gandy et al. 2022 [20]	USA	Prospective cohort	NR	No	8.3 (3–16)	54.5	57.6	CNS tumor	37	Parent	Yes
Irestorm et al. 2022 [21]	Netherlands	Prospective cohort	NR	NR	6.3 (NR)	NR	56.5	ALL	127	Parent	Yes
Jacobs et al. 2022 [22]	USA and Canada	Prospective cohort	Both	NR	13.0 (7–18)	22.2	51.4	LL	257	Patient	Yes
Weaver et al. 2022 [23]	USA	Prospective cohort	Both	No	13.0 (7–17)	43.5	54.7	Mixed^a^	492	Patient	Yes
Wu et al. 2022 [24]	China	Cross‐sectional	Both	NR	8.9 (NR)	NR	60.0	Mixed^a^	40	Patient	No
Bradford et al. 2021 [25]	Australia	Cross‐sectional	Outpatient	No	12 (8–18)	NR	69.0	Mixed^a^	48	Patient	Yes
Cheng et al. 2021 [26]	China	Cross‐sectional	Both	NR	NR	100.0	66.8	Mixed^a^	187	Patient	Yes
Cheng KK et al. 2021 [27]	Singapore	Prospective cohort	Outpatient	Yes	13.7 (10–18)	NR	62.0	Mixed^a^	50	Patient	Yes
Brown et al. 2021 [28]	USA	Cross‐sectional	NR	NR	8.5 (2.6–17.3)	75.0	56.0	ALL	171	Parent and patient	No
Daniel et al. 2020 [29]	USA	Cross‐sectional	Outpatient	NR	10.1 (5–17)	24.5	44.4	Mixed^a^	59	Patient	No
Li et al. 2020 [30]	China	Cross‐sectional	Both	No	8.9 (NR)	NR	61.0	ALL and AML	159	Parent and patient	Yes
Rostagno et al. 2020 [31]	Italy	Prospective cohort	Both	NR	11.7 (5–17)	NR	54.5	Mixed^a^	134	Parent and patient	Yes
Steur et al. 2020 [32]	Netherlands	Prospective cohort	Outpatient	NR	(1–19)	NR	57.0	ALL	151	Parent	No
Cadamuro et al. 2020 [33]	Brazil	Cross‐sectional	Both	Yes	NR	52.2	49.7	Mixed^a^	157	Parent and patient	Yes
Kudubes et al. 2019 [34]	Turkey	Phase 3 RCT	Inpatient	NR	9.4/9.1 (control and experimental group, NR)	NR	57.5	Mixed^a^	80	Parent and patient	Yes
Nagarajan et al. 2019 [35]	USA and Canada	Prospective cohort	Both	No	(2–18)	17.0	52.6	AML	560	Parent and patient	No
Rogers et al. 2019 [36]	USA	Phase 3 RCT	Inpatient	Yes	9.5 (4–19)	18.2	60.6	Medulloblastoma	43	Parent and patient	Yes
Tomlinson et al. 2019 [8]	USA and Canada	Cross‐sectional	Both	Yes	NR	NR	61.3	Mixed^a^	366^b^	Patient	No
Hockenberry et al. 2018 [65]	USA	Prospective cohort	Both	NR	NR	59.1	55.9	ALL	191	Parent and patient	No
Macpherson et al. 2018 [37]	USA	Prospective cohort	Both	No	(8–18)	56.3	54.1	Mixed^a^	96	Patient	Yes
Dobrozsi et al. 2017 [38]	USA	Prospective cohort	Both	NR	11.7 (5–21)	28.0	58.0	Mixed^a^	41	Patient	No
Rodgers et al. 2016 [39]	USA	Prospective cohort	NR	NR	(3–12)	47.0	45.0	ALL	38	Parent and patient	No
Bastani et al. 2015 [40]	Iran	Phase 3 RCT	Inpatient	NR	10.0 (8–12)	NR	68.3	ALL	120	Patient	Yes
Crabtree et al. 2015 [41]	USA	Prospective cohort	NR	NR	7.7 (2–18)	19.0	51.0	Mixed^a^	170	Parent and patient	No
MDR Nunes et al. 2015 [42]	USA	Descriptive with repeated measures	Outpatient	No	12.8 (8–17)	65.7	48.6	Mixed^a^	42	Patient	Yes
Rogers et al. 2014 [43]	USA and Canada	Prospective cohort	Outpatient	NR	8.8 (5–18)	18.3	65.5	ALL	100	Parent and patient	No
Ameringer et al. 2013 [44]	USA	Prospective cohort	Both	NR	15.3 (13–18)	66.7	55.6	Mixed^a^	9	Patient	Yes
Hinds et al. 2013 [45]	USA	Cross‐sectional	Both	NR	12.9 (8–17)	NR	55.5	Mixed^a^	93	Patient	Yes
Wesley et al. 2013 [46]	USA	Cross‐sectional	Both	NR	15.6 (13–19)	38.0	54.9	NR	123	Patient	No
Hooke et al. 2011 [47]	USA	Prospective cohort	NR	NR	(6–17)	NR	66.7	Mixed^a^	30	Patient	No
Miller et al. 2011 [48]	USA	Prospective cohort	Inpatient	NR	13.5 (10–17)	59.0	43.6	Mixed	39	Patient	Yes
Baggott et al. 2010 [5]	USA	Prospective cohort	Both	NR	14.8 (10–18)	NR	51.5	Mixed^a^	66	Patient	Yes
Dupuis et al. 2010 [49]	Canada	Cross‐sectional	Both	NR	9.4 (4–18)	NR	55.0	Mixed^a^	200	Parent	Yes
Erickson et al. 2010 [50]	USA	Prospective cohort	NR	NR	16.1 (12–19)	15.0	50.0	Mixed	20	Patient	Yes
Hockenberry et al. 2010 [51]	USA	Prospective cohort	Both	NR	(7–18)	52.0	57.0	Mixed^a^	67	Parent and patient	No
Walker et al. 2010 [6]	USA	Prospective cohort	Both	NR	14.2 (10–19)	25.0	56.9	Mixed^a^	51	Patient	Yes
Zupanec et al. 2010 [52]	Canada	Cross‐sectional	Outpatient	NR	(4–18)	48.4	79.7	ALL	77	Parent and patient	No
Sitaresmi et al. 2009 [53]	Indonesia	Prospective cohort	Both	NR	7.1 (2–16)	NR	63.0	ALL	51	Parent	Yes
Yeh et al. 2009 [54]	Taiwan	Prospective cohort	Both	NR	14.2 (10–18.9)	NR	58.0	Mixed^a^	144	Patient	Yes
Ekti Genc et al. 2008 [55]	Turkey	Phase 3 RCT	Inpatient	NR	9 (7–12)	NR	61.7	ALL and AML	60	Parent and patient	No
Enskar et al. 2008 [56]	Sweden	Cross‐sectional	Both	No	9.6 (NR)	NR	59.0	Mixed	17	Patient	Yes
Perdikaris et al. 2008 [57]	Greece	Prospective cohort	NR	NR	8.9 (7–12)	NR	65.0	Mixed^a^	40	NR	No
Whitsett et al. 2008 [58]	USA and Canada	Prospective cohort	Inpatient	NR	13.7 (9–17)	8.3	66.7	Mixed	12	Parent and patient	No
Yeh et al. 2008 [59]	Taiwan	Prospective cohort	Inpatient	NR	11.5 (7–17)	NR	54	Mixed^a^	48	Parent and patient	No
Hinds et al. 2007 [60]	USA	Prospective cohort	Inpatient	NR	12.5 (7–18)	28.0	41.0	Mixed^a^	29	Parent, patient and healthcare provider	No
Hinds et al. 2007 [61] (cancer journal paper)	USA and Canada	Prospective cohort	Outpatient	NR	(5–18)	21.0	62.0	ALL	100	Parent and patient	No
Williams et al. 2006 [62]	USA	Cross‐sectional	Inpatient	No	10.4 (2–18)	9.0	45.0	Mixed^a^	11	Patient	Yes

Abbreviations: ALL, acute lymphoblastic leukemia; AML, acute myeloid leukemia; CNS, central nervous system; HCT, hematopoietic stem cell transplant; LL, leukemia and lymphoma; NR, not‐reported; USA, United States of America.

^a^ Mixed: mixed cancer types defined as a category where more than two types of patients with cancer were included.

^b^ Study enrolled 502 patients, of which, 366 were on treatment.

Regarding fatigue assessment, 49% of studies used patient‐reported measures, 11% utilized parent‐reported measures, and 38% employed both measures. Fatigue assessment across the 47 included studies [5, 6, 8, 20, 21, 22, 23, 24, 25, 26, 27, 28, 29, 30, 32, 33, 34, 35, 36, 37, 38, 39, 40, 41, 42, 43, 44, 45, 46, 47, 48, 49, 50, 51, 52, 53, 54, 55, 56, 57, 58, 59, 60, 61, 62, 63, 64] utilized 13 scales (Table S3). The most commonly used scales were the Fatigue Scale‐Child (FS‐C), Fatigue Scale‐Adolescent (FS‐A), or Fatigue Scale‐Parent (FS‐P) in 35% of studies, followed by the Pediatric Quality of Life‐Multidimensional Fatigue Scale (PedsQL‐MFS) in 17% and the Patient‐Reported Outcomes Measurement Information System (PROMIS) scale in 14%.

### 3.2 Study Quality Assessment

Among all studies, 38% had an appropriate sampling frame, 17% had adequate sample size and 36% had suitable study settings (Figure S1). A valid fatigue measurement scale was utilized in 90% of studies, and standard and reliable outcome measurement was employed in 98%. However, blinding was inadequate due to non‐blinded outcome measures. Appropriate statistical methods were used for the overall prevalence analysis in all 26 studies reporting prevalence (Figure S2). Among the 29 studies exploring factors associated with fatigue as a secondary outcome, only 41% adjusted for potential confounders. The response or fatigue assessment rate was deemed adequate in 68% (*N* = 32) of studies, indicating minimal attrition bias.

### 3.3 Primary Outcome

**FIGURE 2:**
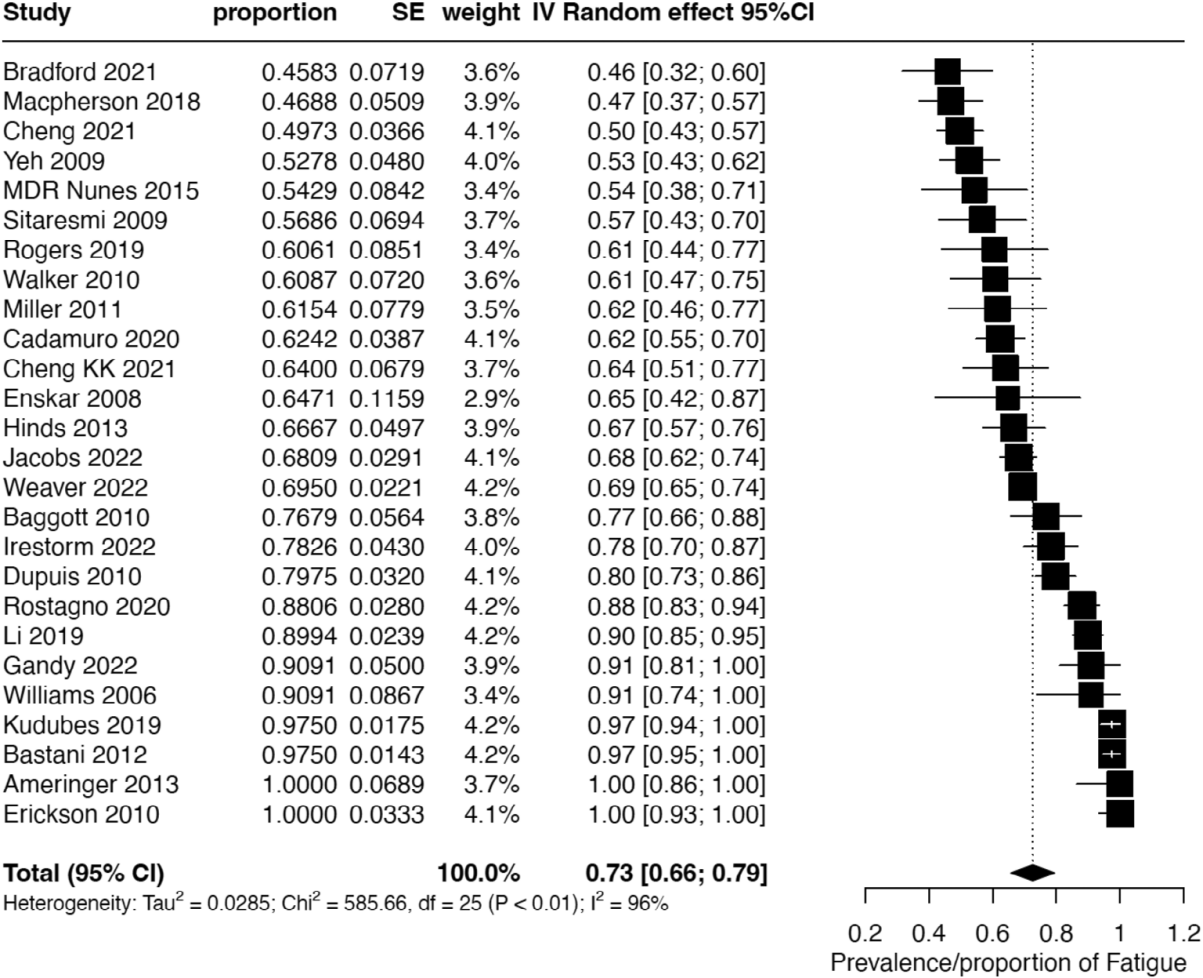
Forest plot of the meta‐analysis of overall fatigue prevalence in included studies (*N* = 26).

In total, 26 studies, comprising 2699 patients (aged 2–18 years), reported the prevalence of overall fatigue, with 2522 patients assessed (Table S4). The reported fatigue prevalence varied from 45.8% to 100.0%. The pooled prevalence estimate across these studies was 73% (95% CI, 66%–79%; *I* ^2^ = 96%) (Figure 2).

Subgroup analysis of overall fatigue prevalence based on predefined factors is detailed in Table 2. Significant differences in fatigue prevalence were observed only in subgroups categorized by treatment setting (*p* < 0.05). Subgroup analyses by age, sex, mood, or anxiety disorder, sleep disorder, and cancer stage were not feasible due to insufficient reporting in the included studies.

**TABLE 2:** Subgroup analysis of the primary outcome of overall prevalence of fatigue.

Subgroup	No. of studies	No. of participants enrolled	Prevalence (%)	95% CI (%)	*I* ^2^ (%)	*p* Value for subgroup difference
**Study type**						0.40
Cross‐sectional	8	872	69	57–81	94	
Prospective cohort	15	1584	72	63–80	93	
Phase 3 RCT	3	243	87	64–100	89	
**Setting**						0.02
Inpatient	5	293	83	66–99	90	
Outpatient	3	140	55	44–66	41	
Both inpatient and outpatient	15	2077	69	61–77	93	
**Type of cancer**						0.58
Mixed cancer types	19	1905	70	62–79	95	
Hematological malignancies	5	714	79	65–93	97	
CNS tumors	2	80	77	47–100	89	
**Type of cancer treatment**						0.38
Chemotherapy	11	1096	78	68–89	95	
Chemotherapy/Surgery/Radiation	4	475	68	47–89	98	
**Type of fatigue scale**						0.48
Childhood Fatigue Scale	4	185	75	50–99	95	
Memorial Symptom Assessment Scale	5	365	72	60–84	88	
Symptom Screening in Pediatrics Tool	2	205	55	39–71	76	
PROMIS Fatigue Subscale	4	1029	64	55–73	87	
PedsQL Multidimensional Fatigue Scale	4	411	69	52–87	94	
**Person reporting fatigue**						0.33
Parent/Caregiver	4	415	77	64–90	81	
Patient	17	1711	69	60–78	96	
Patient and parent/Caregiver	5	573	81	66–95	95	
**Adequate sample frame**						0.95
Yes	11	1751	71	64–79	91	
Unclear	12	829	73	61–86	97	
No	3	119	73	45–100	94	
**Adequate sampling**						0.78
Yes	11	1033	73	64–83	92	
Unclear	11	1206	74	63–85	97	
No	4	460	66	45–87	97	
**Adequate response rate**						0.90
Yes	17	1388	74	65–83	96	
Unclear	3	425	71	42–100	98	
No	6	886	71	61–80	88	

Abbreviations: CI, confidence interval; CNS, central nervous system; PedsQL, Pediatric Quality of Life; PROMIS, Patient‐Reported Outcomes Measurement Information System; RCT, randomized controlled trial.

### 3.4 Secondary Outcomes

#### 3.4.1 Severe Fatigue

**FIGURE 3:**
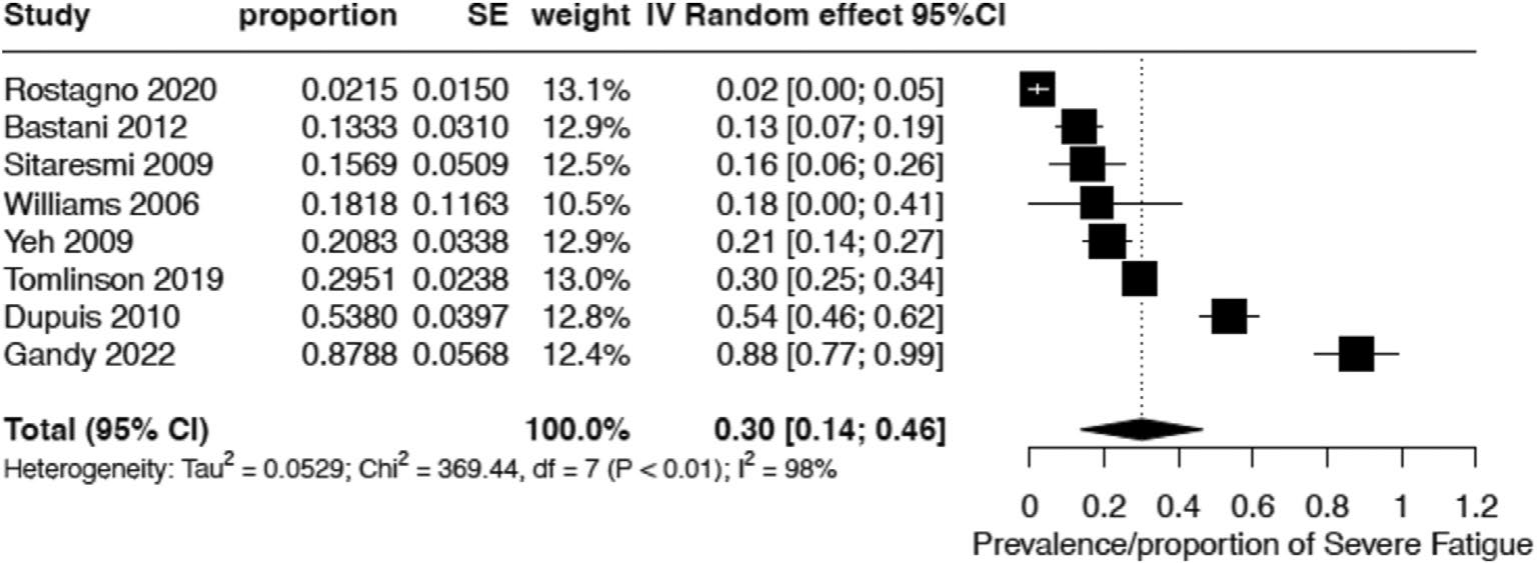
Forest plot of pooled prevalence of severe fatigue (*N* = 8).

Severe fatigue prevalence was reported in 8 (17%) studies, encompassing 1027 patients (Figure 3 and Table S3) [20, 31, 40, 49, 53, 54, 62, 63]. These studies utilized six fatigue scales, with PedsQL‐MFS being the most frequently employed (25%, *N* = 2). Among the eight studies, 5 (62%) included patients with different cancer diagnoses. The pooled prevalence of severe fatigue across these studies was 30% (95% CI 14%–46%, *I* ^2^ = 98%).

#### 3.4.2 Factors Associated With Fatigue

Twenty‐nine studies reported potential factors associated with fatigue among children and adolescents with cancer during active cancer treatment (Table 3). Meta‐analysis for this outcome was not conducted due to the considerable heterogeneity in the methods used to analyze and report (e.g., correlation, univariable, or multivariable analysis) and the study populations; therefore, data on the factors associated with fatigue are presented descriptively: (a) Demographic factors: Among six studies, three found older age associated with higher fatigue older age and higher fatigue [8, 22, 35, 36], two found no association [28, 60], and one found younger age associated with higher fatigue [38]. Seven studies examined the association of sex/gender with fatigue, with five finding no association and two identifying higher fatigue in females [22, 57]. Four studies explored race/ethnicity, with one finding Hispanic patients reporting higher fatigue, while the other three found no such association [22, 28, 35, 46]; (b) disease‐related factors: One study found that patients with CNS/solid tumors experienced less fatigue than those with leukemia/lymphoma [38], while another found no association with the type of leukemia/lymphoma [22]. Two studies in acute lymphoblastic leukemia (ALL) risk grouping showed mixed results, with one finding no association and the other reporting less fatigue in high‐risk and very high‐risk ALL patients compared to low and average‐risk patients [21, 28]; (c) treatment‐related factors: Among brain tumor patients undergoing radiotherapy, cranial spinal radiation dose was a significant predictor of fatigue in one study [20]. Dexamethasone pulse phases and steroid use during chemotherapy were associated with higher fatigue in ALL patients [32, 59]. In acute myeloid leukemia patients, the number of CTCAE toxicities was significantly associated with general fatigue [35]. Fatigue varied during treatment, decreasing over time in ALL patients and during the initial weeks of leukemia/lymphoma treatment [5, 41, 65]; (d) Sleep‐related factors: Seven studies found that poor sleep measures, such as inconsistent sleep habits, fragmented sleep, high caregiver‐reported sleep problems, frequent night awakenings and high nighttime activity, were associated with higher fatigue [32, 36, 44, 52, 60, 68, 69]; (e) Other clinical and psychosocial symptoms: Two studies found a positive association between fatigue and pain [26, 46] and two studies found a positive correlation between depressive symptoms and fatigue [44, 51, 58].

**TABLE 3:** Factors associated with fatigue in the individual study included (*N* = 29).

Study ID	Type of cancer	Factors analyzed	Type of analysis	Factors Associated/Correlated with greater fatigue	Factors Associated/Correlated with lower fatigue
Gandy et al. 2022 [20]	CNS tumor	Radiotherapy (proton vs. photon), hydrocephalus (None vs. Any), craniospinal radiation, age at radiation therapy	Multivariable regression analysis	Craniospinal radiation during first week of therapy	None
Irestorm et al. 2022 [21]	ALL	Couse of fatigue, sex, risk group at diagnosis, serious adverse event	Multivariable regression analysis	Course of fatigue during treatment predicted fatigue reported at follow‐up	None
Jacobs et al. 2022 [22]	Leukemia and lymphoma	Gender, age, ethnicity, time since diagnosis, diagnostic group (ALL/AML/NHL/HL), and caregiver's education level	Mixed effects model	Female, Hispanic patients	Male, older age, non‐Hispanic
Wu et al. 2022 [24]	Mixed	Quality‐of‐life distress	Multivariable regression analysis	Quality‐of‐ life distress	None
Cheng et al. 2021 [26]	Mixed	Pain interference, depression and lower mobility	Multivariable regression analysis	Greater pain interference, depressive symptoms and less mobility	None
Brown et al. 2021 [28]	ALL	Age at diagnosis, BMI, gender, race/ethnicity, type of leukemia, CNS involvement at diagnosis, high‐or very high‐risk ALL, and asparagine and gamma‐glutamyl glutamine in CSF	Multivariable regression analysis	Low‐risk and average‐risk ALL, asparagine and gamma‐glutamyl glutamine in CSF	High‐risk and very‐high risk ALL
Daniel et al. 2021 [29]	Mixed	Sleep timing, sleep consistency, technology use, presence of someone else, sleep disturbance, sleep‐related impairment, pain interference, nausea and sleep consistency	Correlation and Simple mediation models	Sleep disturbances, Sleep‐related impairment, pain interference and nausea	Consistent caregiver‐reported sleep routines
Steur et al. 2020 [32]	ALL	Dexamethasone pulses during maintenance chemotherapy, sleep–wake rhythm, stable sleep–wake rhythm, robust sleep–wake rhythm, more physical activity during the day and fragmented sleep–wake rhythm	Multivariable regression analysis	Dexamethasone pulses, fragmented sleep–wake rhythm	Robust sleep–wake rhythm, less fragmented sleep and higher physical activity during dexamethasone‐free periods
Nagarajan et al. 2019 [35]	AML	Age, sex, White race, Hispanic ethnicity, insurance status, high‐risk AML, bortezomib arm assignment, days of neutropenia, and number of submitted CTCAE toxicities	Multivariable regression analysis	Older age and number of submitted CTCAE toxicities	None
Rogers et al. 2019 [36]	Medulloblastoma	Age, percent sleep, longest sleep episode and nighttime activity score on actigraphy. Amplitude, 24 h auto‐correlation, intra‐daily variability, inter‐daily stability, dichotomy index on actigraphy	Linear mixed models and correlation	Higher age, lower percent nighttime sleep and higher nighttime activity scores (adolescent‐reported fatigue), longest nighttime sleep episode (child‐reported fatigue), dysregulated amplitude, 24 h auto‐correlation, and intra‐daily variability	Lower age
Hockenberry et al. 2018 [65]	ALL	3NT (protein 3‐nitrotyrosine) in CSF and time since induction treatment	Latent class growth analysis and mixed models	Higher 3NT in CSF	Time from one treatment phase to another when measured from post‐induction to 12 months post‐induction chemotherapy
Dobrozsi et al. 2017 [38]	Mixed	Age, gender, type of cancer, time since diagnosis, and intensity of therapy	Linear mixed models	Leukemia/lymphoma	Older age and diagnosis of solid/CNS tumors
Rodgers et al. 2016 [39]	ALL	Reduced glutathione (GSH) and reduced/oxidized glutathione (GSH/GSSG) ratio in the CSF	Correlation	Low mean GSH/GSSG ratios in CSF	None
Crabtree et al. 2015 [41]	Mixed	Type of cancer, gender, age, and socioeconomic status, steroid use, radiation and chemotherapy, insomnia, sleep hygiene, bedtime, wake time, total sleep time, or restless sleep within 30 days of diagnosis and 8 weeks later	Univariable analysis and multivariable regression analysis	Longer sleep duration (6–12‐year‐old)	Younger children with leukemia/lymphoma had a significant decline in parent‐reported fatigue within 30 days of diagnosis and 8 weeks later than those with solid tumor/CNS tumors
MDR Nunes et al. 2015 [42]	Mixed	Age, gender, cancer type, sleep duration	Univariable analysis and correlation	Adolescents, females, sarcoma, less sleep duration	Younger children, males
Rogers et al. 2014 [43]	ALL	Circadian activity rhythm parameters—peak, midline estimating statistic of rhythm (MESOR), amplitude, acrophase and circadian quotient	Linear mixed models	None	Peak activity, MESOR and amplitude
Ameringer et al. 2013 [44]	Mixed	Anxiety and sleep disturbances	Correlation	Disturbed sleep	Higher trait anxiety
Wesley et al. 2013 [46]	NR	Age, gender, minority status, pain, nausea, positive and negative affect, stressful life events, family support, friend support and family functioning	Correlation	Pain, nausea, and positive affect	Negative affect
Hooke et al. 2011 [47]	Mixed	Gender, type of cancer and time since first three cycles of chemotherapy	Univariable analysis	None	For young children, fatigue significantly decreased during the first three cycles of chemotherapy, and the ALL group had a greater decrease in fatigue than the lymphoma or the solid tumor group from cycle 1 to cycle 3 of chemotherapy
Baggott et al. 2010 [5]	Mixed	Time since administration of a chemotherapy cycle	Multilevel logistic regression analysis	None	For each week following a cycle of chemotherapy, odds of reporting fatigue were lower than the previous week for 2 weeks after the initiation of chemotherapy cycle
Hockenberry et al. 2010 [51]	Mixed	Depression on day 7 of chemotherapy	Correlation	Depressive symptoms	None
Zupanec et al. 2010 [52]	ALL	Perceived problems in sleep, different sleep since diagnosis, different sleep place, sleep in 20 min, moving to different bed in the night and duration of weeknight sleep	Correlation	Sleep problems (4–12 years), Different sleep since diagnosis (4–7 years), moving to another bed (4–12 years)	None
Ekti Genc et al. 2008 [55]	ALL & AML	Sex, diagnosis, age, hemoglobin, mucositis, nausea and vomiting	Correlation	None	None
Enskar et al. 2008 [56]	Mixed	Life satisfaction	Univariable analysis	Less life satisfaction	None
Perdikaris et al. 2008 [57]	Mixed	Gender	Multivariable regression analysis	Females	None
Whitsett et al. 2008 [58]	Mixed	Depression	Correlation	Depression	None
Yeh et al. 2008 [66]	Mixed	Steroid use prior to start of chemotherapy cycle, steroids use per day of cycle, hemoglobin value, prior chemotherapy, cumulative doses of chemotherapy drugs in the cycle	Multivariable analysis	Steroids used before chemotherapy cycle, hemoglobin value, steroid use for each day of chemotherapy cycle and prior chemotherapy	None
Hinds et al. 2007 [60]	Mixed	Number of nocturnal awakenings during inpatient hospital stay, age, diagnosis, gender, baseline fatigue, or length of hospitalization, hematocrit and hemoglobin level	Mixed effect model	More night awakenings (20 or more)	None
Hinds et al. 2007 [61]	Mixed	Dexamethasone treatment, age, sex and ALL risk group	Correlation	Dexamethasone treatment	None

Abbreviations: ALL, acute lymphoblastic leukemia; AML, acute myeloid leukemia; CNS; central nervous system; CSF, cerebrospinal fluid; CTCAE, common terminology criteria for adverse events; HL, Hodgkin lymphoma; LSS‐C, Life Situation Scale for Children; NHL, non‐Hodgkin lymphoma.

### 3.5 Publication Bias

Visual inspection of the funnel plot exploring the publication bias of the studies contributing to the over fatigue prevalence showed that smaller studies reporting higher fatigue prevalence were underrepresented (Figure S3). Egger's regression test, assessing asymmetry in the funnel plot, demonstrated a statistically significant result (*t* = −3.77 and *p*—0.0009), indicating the presence of publication bias.

## 4 Discussion

In this review, we found high heterogeneity in summary effect measures, with the pooled prevalence of overall fatigue at 73% and severe fatigue at 30% in children and adolescents undergoing cancer treatment. These findings are comparable to a recent review demonstrating a prevalence of 62% among adults with cancer during treatment [70]. The high fatigue prevalence in our population also aligns with reports of increased fatigue among children and adolescents with chronic diseases such as cystic fibrosis and autoimmune diseases [71, 72]. Notably, this prevalence is seven times higher than the 10.1% prevalence of general fatigue among adolescents in the general population [73].

Stratified analyses by treatment setting revealed a lower prevalence among patients receiving outpatient treatment than those getting treatment either as an inpatient or both inpatient and outpatient. Typically, inpatient cancer treatments are more intensive than outpatient chemotherapy; plausibly, fatigue is more prevalent during inpatient chemotherapy cycles due to the increased intensity of therapy, increased distress associated with inpatient admissions impacting the psychological aspects of fatigue, fewer interactions with family and less time spent being physically active and more sleep disruptions due to night awakenings, and the noisy environment from the monitors [60, 74, 75, 76]. Additional studies are warranted to ascertain if outpatient delivery of a similar chemotherapy regimen leads to lesser fatigue than inpatient delivery.

Studies reporting on fatigue‐related factors examined various demographic, disease‐related, treatment‐related, and psychological factors but revealed inconsistent findings due to substantial heterogeneity. However, most studies found consistent associations between sleep patterns and fatigue, with stable sleep habits linked to lower fatigue levels. The literature indicates a reciprocal and strong relationship between cancer‐related fatigue and sleep disturbances through shared physiological pathways [77, 78]. Future research should target pediatric‐specific interventions to improve fatigue and sleep quality in children and adolescents with cancer.

Most studies demonstrated a positive association between pain, depression, and fatigue, in addition to sleep. This supports the concept of symptom clustering in oncology, an evolving concept that underscores the importance of clinicians recognizing the co‐occurrence of symptoms and the need to address multiple symptoms within a cluster to achieve better overall symptom control [51, 66].

Despite its heterogeneity, this systematic review's primary strength is in systematically presenting data on the prevalence of fatigue and associated factors among children and adolescents with cancer during treatment based on multiple studies conducted to date. A comprehensive search of several databases from their inception, utilizing broad search terms and robust methodology, was performed to avoid bias. Subgroup analyses were conducted to explore the causes of heterogeneity systematically. Finally, a multidisciplinary team provided expertise in conducting the review and interpreting the findings.

This review has several notable limitations. The included studies exhibited substantial heterogeneity in patient demographics, cancer diagnoses, treatment modalities, fatigue assessment tools, and follow‐up durations. Consequently, despite pooling the prevalence data, our confidence in the estimated pooled prevalence remains low. The secondary objective of identifying factors associated with fatigue also suffered from inconsistent adjustment for potential confounders across studies. Only 3 out of 27 studies scored well on all the domains of study quality assessment for external validity, with 60% failing to specify sampling methods, thus raising concerns about selection bias. The reliance on self‐reported fatigue questionnaires precluded participant blinding, resulting in high detection bias. Additionally, variations in fatigue prevalence estimates may result from inconsistent reporting sources, such as caregiver versus child self‐reports. Caregiver/proxy reports may overestimate child‐reported fatigue, leading to discrepancies in prevalence estimates among studies [79]. Fatigue, a multifaceted construct, was assessed in this review as a general or total measure, limiting our ability to comment on specific dimensions of fatigue. The restriction to English‐language studies may have introduced publication bias, and the exclusion of studies focusing on children and adolescents with relapsed/refractory cancer or those receiving palliative care limits the applicability of the findings to these populations.

## 5 Conclusions

In conclusion, our systematic review and meta‐analysis reveal that 73% of children and adolescents undergoing cancer treatment experience fatigue, with nearly 30% enduring severe fatigue. Despite the significant heterogeneity among studies, indicating a low certainty in these estimates, the high prevalence and variable nature of fatigue during treatment are evident. The high prevalence and significant impact of fatigue on the quality of life and other critical aspects of life for children and adolescents with cancer underscore the need for routine assessment using valid, reliable, and psychometrically robust fatigue scales in clinical settings [3, 80].

Since effective interventions such as physical activity, mindfulness, and relaxation interventions to mitigate fatigue even in younger children are available, it is paramount that healthcare professionals should not consider fatigue an inevitable toxicity of treatment and utilize effective approaches to address it with their patients [81, 82, 83]. Future research should focus on identifying patterns and predictors of fatigue persistence beyond treatment, employing consistent assessment methods and multivariable analyses while ensuring the inclusion of younger children to address current gaps.

## Author Contributions

**Sapna Oberoi:** conceptualization (lead), data curation (lead), formal analysis (lead), investigation (lead), methodology (lead), project administration (lead), resources (lead), visualization (lead), writing – original draft (lead), writing – review and editing (lead). **Beili Huang:** conceptualization (supporting), data curation (supporting), formal analysis (supporting), project administration (supporting), writing – review and editing (supporting). **Rasheda Rabbani:** conceptualization (supporting), formal analysis (equal), investigation (supporting), methodology (equal), software (lead), visualization (equal), writing – review and editing (supporting). **Nicole Askin:** data curation (supporting), investigation (supporting), methodology (supporting), resources (supporting), writing – review and editing (supporting). **George Okoli:** data curation (supporting), formal analysis (supporting), investigation (supporting), methodology (supporting), project administration (supporting), resources (supporting), writing – review and editing (supporting). **Richa Jain:** data curation (supporting), formal analysis (supporting), investigation (supporting), project administration (supporting), writing – review and editing (supporting). **Lillian Sung:** conceptualization (supporting), investigation (supporting), methodology (supporting), supervision (supporting), writing – review and editing (supporting). **Maya M. Jeyaraman:** conceptualization (supporting), formal analysis (supporting), investigation (supporting), methodology (supporting), supervision (supporting), writing – review and editing (supporting). **Alyson Mahar:** conceptualization (supporting), formal analysis (supporting), investigation (supporting), investigation (supporting), methodology (supporting), methodology (supporting), project administration (supporting), project administration (supporting), supervision (equal), supervision (equal), writing – original draft (supporting), writing – original draft (supporting), writing – review and editing (supporting), writing – review and editing (supporting). **Roberta Woodgate:** conceptualization (supporting), formal analysis (supporting), investigation (supporting), methodology (supporting), project administration (supporting), supervision (equal), writing – original draft (supporting), writing – review and editing (supporting). **Ryan Zarychanski:** conceptualization (equal), formal analysis (supporting), investigation (supporting), methodology (equal), project administration (equal), supervision (lead), writing – original draft (supporting), writing – review and editing (supporting).

## Ethics Statement

This study is exempt from ethics approval as it is a systematic review that utilized data from previously published data from trials.

## Conflicts of Interest

The authors declare no conflicts of interest.

## Supporting information


Data S1.

## Data Availability

The data supporting this study's findings are available from the corresponding author upon reasonable request.
