# Supplementary material for: Prevalence and Factors Associated With Cancer‐Related Fatigue Among Children and Adolescents Undergoing Cancer Treatment: A Systematic Review and Meta‐Analysis
Source: Cancer Med. 2024 Dec 11;13(23):e70502. doi: 10.1002/cam4.70502 (PMC11632266; doi:10.1002/cam4.70502)
Supplement: Supplementary file 1 — Data S1. [file CAM4-13-e70502-s001.docx]

**APPENDICES**

**TITLE: PREVALENCE AND FACTORS ASSOCIATED WITH CANCER-RELATED FATIGUE AMONG CHILDREN AND ADOLESCENTS UNDERGOING CANCER TREATMENT: A SYSTEMATIC REVIEW AND META-ANALYSIS**

**Table S1. Search Strategy**

| **Medline Search Strategy**  exp neoplasms/ or cancer survivors/  (cancer* or neoplas* or tumo?r* or malignan* or metasta* or oncogen* or oncolog*).tw,kf.  (leukemia* or leukaemia* or leukocythemia* or leukocythaemia* or childhood ALL or AML or JMML or JCML or lymphoma* or hodgkin* or nonhodgkin* or burkitt* or nonburkitt* or blastoma* or neuroblastoma* or meningioma* or wilm or wilms or nephroblastoma* or rhabdomyosarcoma* or myosarcoma* or sarcoma* or osteosarcoma* or ewing* or retinoblastoma* or teratoma* or HCC or hepatocellular carcinoma* or hepatic carcinoma* or hepatoblastoma* or hepatoma* or pnet or medulloblastoma* or ganglioma* or glioma* or atrt or pancreatoblastoma* or glioblastoma* or infantile fibrosarcoma* or egct or jgct or ependymoma* or pxa or xanthoastrocytoma* or oligodendroglioma* or astrocytoma* or choroid plexus papilloma* or choroid plexus carcinoma* or dipg or gliofibroma* or pmmti or dscrct or synovial sarcoma*).tw,kf.  or/1-3  exp pediatrics/ or exp pediatricians/ or exp nurses, pediatric/ or pediatric nurse practitioners/ or pediatric assistants/ or adolescent medicine/ or hospitals, pediatric/ or exp pediatric nursing/ or exp intensive care units, pediatric/ or intensive care, neonatal/ or exp infant/ or exp child/ or adolescent/ or child health/ or infant health/ or adolescent health/ or exp child health services/ or adolescent health services/  (p?ediatric* or infan* or baby* or babies or neonat* or newborn* or new-born* or child* or kid or kids or schoolchild* or school age* or schoolage* or primary school* or elementary school* or primary age* or elementary age* or secondary school* or high school* or highschool* or preschool* or pre-school* or nursery or toddler* or kindergar* or adoles* or teen* or youth or youths or young people or young person* or young adult* or preteen* or pre-teen* or pubescen* or prepubescen* or pre-pubescen* or boy* or girl* or nicu or picu or juvenile* or preadolescen*).tw,kf.  or/5-6  fatigue/ or mental fatigue/ or muscle fatigue/ or lethargy/ or sleepiness/ or exp physical endurance/ or asthenia/  (fatigu* or tired* or tire or weary or weariness or wearied or endurance or exhaustion or exhausted or letharg* or listless* or sleepy or sleepiness or drowsy or drowsiness or somnolen* or astheni* or ((reduced or loss or low or diminished or less) adj2 (energy or vitality)) or lassitude).tw,kf.  or/8-9  exp epidemiologic studies/  (case control? or case comparison? or case base? or case referent or case referrent or case compeer or matched case?).tw,kf.  (cohort adj (study or studies or analy$)).tw,kf.  interrupted time series.tw,kf.  (control* adj2 (before-after or "before and after")).tw,kf.  (historic* adj2 control*).tw,kf.  ((Follow up or followup) adj (study or studies)).tw,kf.  ((observation* or epidemiologic* or comparative or comparison) adj (study or studies)).tw,kf.  (longitudinal or retrospective or prospective).tw,kf.  (Cross section* or crosssection*).tw,kf.  (comparative study or observational study).pt.  Randomized Controlled Trials as Topic/  randomized controlled trial/  Random Allocation/  Double Blind Method/  Single Blind Method/  clinical trial/  clinical trial, phase ii.pt.  clinical trial, phase iii.pt.  clinical trial, phase iv.pt.  controlled clinical trial.pt.  multicenter study.pt.  (clinical trial or pragmatic clinical trial).pt.  exp Clinical Trials as topic/  (clinical adj trial$).tw,kf.  (RCT or RCTs or non-RCT or non-RCTs or non-random* or quasi-random* or quasi-experimental*).tw,kf.  ((singl$ or doubl$ or treb$ or tripl$) adj (blind$3 or dumm* or mask$3)).tw,kf.  PLACEBOS/  (placebo$ or sham).tw,kf.  (randomized or randomised or randomly).ab.  trial.ti.  or/11-41  4 and 7 and 10 and 42 =2383  **Embase Search Strategy**  exp neoplasm/ or cancer survivor/  (cancer* or neoplas* or tumo?r* or malignan* or metasta* or oncogen* or oncolog*).tw,kw.  (leukemia* or leukaemia* or leukocythemia* or leukocythaemia* or childhood ALL or AML or JMML or JCML or lymphoma* or hodgkin* or nonhodgkin* or burkitt* or nonburkitt* or blastoma* or neuroblastoma* or meningioma* or wilm or wilms or nephroblastoma* or rhabdomyosarcoma* or myosarcoma* or sarcoma* or osteosarcoma* or ewing* or retinoblastoma* or teratoma* or HCC or hepatocellular carcinoma* or hepatic carcinoma* or hepatoblastoma* or hepatoma* or pnet or medulloblastoma* or ganglioma* or glioma* or atrt or pancreatoblastoma* or glioblastoma* or infantile fibrosarcoma* or egct or jgct or ependymoma* or pxa or xanthoastrocytoma* or oligodendroglioma* or astrocytoma* or choroid plexus papilloma* or choroid plexus carcinoma* or dipg or gliofibroma* or pmmti or dscrct or synovial sarcoma*).tw,kw.  or/1-3  exp pediatrics/ or exp pediatrician/ or pediatric ward/ or neonatologist/ or pediatric nurse/ or neonatal nurse/ or exp pediatric nursing/ or exp newborn nursing/ or pediatric nurse practitioner/ or neonatal nurse practitioner/ or pediatric hospital/ or newborn intensive care/ or exp child/ or exp adolescent/ or child health/ or adolescent health/ or exp child health care/  (p?ediatric* or infan* or baby* or babies or neonat* or newborn* or new-born* or child* or kid or kids or schoolchild* or school age* or schoolage* or primary school* or elementary school* or primary age* or elementary age* or secondary school* or high school* or highschool* or preschool* or pre-school* or nursery or toddler* or kindergar* or adoles* or teen* or youth or youths or young people or young person* or young adult* or preteen* or pre-teen* or pubescen* or prepubescen* or pre-pubescen* or boy* or girl* or nicu or picu or juvenile* or preadolescen*).tw,kw.  or/5-6  exp fatigue/ or lethargy/ or somnolence/ or endurance/ or asthenia/ or drowsiness/ or listlessness/  (fatigu* or tired* or tire or weary or weariness or wearied or endurance or exhaustion or exhausted or letharg* or listless* or sleepy or sleepiness or drowsy or drowsiness or somnolen* or astheni* or ((reduced or loss or low or diminished or less) adj2 (energy or vitality)) or lassitude).tw,kw.  or/8-9  clinical study/ or case control study/ or family study/ or longitudinal study/ or retrospective study/ or prospective study/ or cohort analysis/  (case control? or case comparison? or case base? or case referent or case referrent or case compeer or matched case?).ti,ab,kw.  (cohort adj (study or studies or analy$)).ti,ab,kw.  interrupted time series.ti,ab,kw.  (control* adj2 (before-after or "before and after")).ti,ab,kw.  (historic* adj2 control*).ti,ab,kw.  ((Follow up or followup) adj (study or studies)).ti,ab,kw.  ((observation* or epidemiologic* or comparative or comparison) adj (study or studies)).ti,ab,kw.  (longitudinal or retrospective or prospective).ti,ab,kw.  (Cross section* or crosssection*).ti,ab,kw.  clinical trial/  randomized controlled trial/  controlled clinical trial/  multicenter study/  exp randomization/  Double Blind Procedure/  Single Blind Procedure/  Phase 2 clinical trial/  Phase 3 clinical trial/  Phase 4 clinical trial/  crossover procedure/  (RCT or RCTs or quasi-random* or quasi-experimental* or (random$ adj2 allocat$)).ti,ab,kw.  ((singl$ or doubl$ or trebl$ or tripl$) adj (blind$3 or dumm* or mask$3)).ti,ab,kw.  PLACEBO/  (placebo$ or sham).ti,ab,kw.  (randomized or randomised or randomly).ab.  trial.ti.  or/11-37  4 and 7 and 10 and 38 =5920  **CINAHL Search Strategy**  (MH neoplasms+) or (MH cancer survivors)  (cancer* or neoplas* or tumo?r* or malignan* or metasta* or oncogen* or oncolog*)  (leukemia* or leukaemia* or leukocythemia* or leukocythaemia* or "childhood ALL" or AML or JMML or JCML or lymphoma* or hodgkin* or nonhodgkin* or burkitt* or nonburkitt* or blastoma* or neuroblastoma* or meningioma* or wilm or wilms or nephroblastoma* or rhabdomyosarcoma* or myosarcoma* or sarcoma* or osteosarcoma* or ewing* or retinoblastoma* or teratoma* or HCC or (hepatocellular N1 carcinoma*) or (hepatic N1 carcinoma*) or hepatoblastoma* or hepatoma* or pnet or medulloblastoma* or ganglioma* or glioma* or atrt or pancreatoblastoma* or glioblastoma* or (infantile N1 fibrosarcoma*) or egct or jgct or ependymoma* or pxa or xanthoastrocytoma* or oligodendroglioma* or astrocytoma* or ("choroid plexus" N1 papilloma*) or ("choroid plexus" N1 carcinoma*) or dipg or gliofibroma* or pmmti or dscrct or (synovial N1 sarcoma*))  S1 OR S2 OR S3  (MH pediatrics+) or (MH pediatricians) or (MH neonatologists) or (MH "pediatric nurse practitioners+") or (MH "adolescent medicine") or (MH "hospitals, pediatric") or (MH "pediatric nursing+") or (MH "pediatric units+") or (MH child+) or (MH adolescence+) or (MH "child health") or (MH "adolescent health") or (MH "child health services+") or (MH "adolescent health services")  (pediatric* or paediatric* or infan* or baby* or babies or neonat* or newborn* or new-born* or child* or kid or kids or schoolchild* or (school N1 age*) or schoolage* or (primary N1 school*) or (elementary N1 school*) or (primary N1 age*) or (elementary N1 age*) or (secondary N1 school*) or (high N1 school*) or highschool* or preschool* or pre-school* or nursery or toddler* or kindergar* or adoles* or teen* or youth or youths or "young people" or (young N1 person*) or (young N1 adult*) or preteen* or pre-teen* or pubescen* or prepubescen* or pre-pubescen* or boy* or girl* or nicu or picu or juvenile* or preadolescen*)  S5 OR S6  (MH fatigue+) or (MH "muscle fatigue") or (MH sleepiness) or (MH "physical endurance+") or (MH asthenia)  (fatigu* or tired* or tire or weary or weariness or wearied or endurance or exhaustion or exhausted or letharg* or listless* or sleepy or sleepiness or drowsy or drowsiness or somnolen* or astheni* or ((reduced or loss or low or diminished or less) N2 (energy or vitality)) or lassitude)  S8 OR S9  (MH "prospective studies+") or (MH "case control studies+") or (MH "correlational studies") or (MH "cross sectional studies") or (MH "controlled before-after studies") or (MH "historically controlled study") or (MH "interrupted time series analysis") or (MH "nonrandomized trials")  ("case control" or "case comparison" or "case base" or "case based" or "case referent" or "case referrent" or "case compeer" or "matched case")  (cohort N1 (study or studies or analy*))  "interrupted time series"  control* N2 (before-after or "before and after")  historic* N2 control*  (("Follow up" or followup) N1 (study or studies))  ((observation* or epidemiologic* or comparative or comparison) N1 (study or studies))  (longitudinal or retrospective or prospective)  (Cross section* or crosssection*)  (MH "clinical trials+")  PT "clinical trial"  MH "Random Assignment"  (MH "Double-Blind Studies") OR (MH "Single-Blind Studies") OR (MH "Triple-Blind Studies")  TX clinic* N1 trial*  TX randomi* control* trial*  (RCT or RCTs or quasi-random* or quasi-experimental*)  TX (singl* or doubl* or trebl* or tripl*) N1 (blind* or dumm* or mask*)  (MH placebos)  (TX placebo) or (TX sham)  AB (randomized or randomised or randomly)  TI trial  TX allocat* N2 random*  S11 OR S12 OR S13 OR S14 OR S15 OR S16 OR S17 OR S18 OR S19 OR S20 OR S21 OR S22 OR S23 OR S24 OR S25 OR S26 OR S27 OR S28 OR S29 OR S30 OR S31 OR S32 OR S33  S4 and S7 and S10 and S34 =1705  **PsychINFO Search Strategy**  exp neoplasms/  (cancer* or neoplas* or tumo?r* or malignan* or metasta* or oncogen* or oncolog*).ti,ab,id.  (leukemia* or leukaemia* or leukocythemia* or leukocythaemia* or childhood ALL or AML or JMML or JCML or lymphoma* or hodgkin* or nonhodgkin* or burkitt* or nonburkitt* or blastoma* or neuroblastoma* or meningioma* or wilm or wilms or nephroblastoma* or rhabdomyosarcoma* or myosarcoma* or sarcoma* or osteosarcoma* or ewing* or retinoblastoma* or teratoma* or HCC or hepatocellular carcinoma* or hepatic carcinoma* or hepatoblastoma* or hepatoma* or pnet or medulloblastoma* or ganglioma* or glioma* or atrt or pancreatoblastoma* or glioblastoma* or infantile fibrosarcoma* or egct or jgct or ependymoma* or pxa or xanthoastrocytoma* or oligodendroglioma* or astrocytoma* or choroid plexus papilloma* or choroid plexus carcinoma* or dipg or gliofibroma* or pmmti or dscrct or synovial sarcoma*).ti,ab,id.  or/1-3  pediatrics/ or pediatricians/ or neonatal period/ or early adolescence/ or neonatal intensive care/  ("100" or "200").ag.  (p?ediatric* or infan* or baby* or babies or neonat* or newborn* or new-born* or child* or kid or kids or schoolchild* or school age* or schoolage* or primary school* or elementary school* or primary age* or elementary age* or secondary school* or high school* or highschool* or preschool* or pre-school* or nursery or toddler* or kindergar* or adoles* or teen* or youth or youths or young people or young person* or young adult* or preteen* or pre-teen* or pubescen* or prepubescen* or pre-pubescen* or boy* or girl* or nicu or picu or juvenile* or preadolescen*).ti,ab,id.  or/5-7  fatigue/ or sleepiness/ or physical endurance/ or exp asthenia/  (fatigu* or tired* or tire or weary or weariness or wearied or endurance or exhaustion or exhausted or letharg* or listless* or sleepy or sleepiness or drowsy or drowsiness or somnolen* or astheni* or ((reduced or loss or low or diminished or less) adj2 (energy or vitality)) or lassitude).ti,ab,id.  or/9-10  cohort analysis/ or followup studies/ or exp longitudinal studies/ or retrospective studies/ or between groups design/  ("0430" or "0450" or "0451" or "0453").md.  (case control? or case comparison? or case base? or case referent or case referrent or case compeer or matched case?).ti,ab,id.  (cohort adj (study or studies or analy$)).ti,ab,id.  interrupted time series.ti,ab,id.  (control* adj2 (before-after or "before and after")).ti,ab,id.  (historic* adj2 control*).ti,ab,id.  ((Follow up or followup) adj (study or studies)).ti,ab,id.  ((observation* or epidemiologic* or comparative or comparison) adj (study or studies)).ti,ab,id.  (longitudinal or retrospective or prospective).ti,ab,id.  (Cross section* or crosssection*).ti,ab,id.  clinical trials/ or between groups design/  0300.md.  (clinical adj trial$).ti,ab,id.  (RCT or RCTs or quasi-random* or quasi-experimental* or (random$ adj2 allocat$)).ti,ab,id.  ((singl$ or doubl$ or trebl$ or tripl$) adj (blind$3 or dumm* or mask$3)).ti,ab,id.  PLACEBO/  (placebo$ or sham).ti,ab,id.  (randomized or randomised or randomly).ab.  trial.ti.  or/12-31  4 and 8 and 11 and 32 =186  **CENTRAL Search Strategy**  [mh neoplasms] or [mh ^"cancer survivors"]  (cancer* or neoplas* or tumo?r* or malignan* or metasta* or oncogen* or oncolog*):ti,ab,kw  (leukemia* or leukaemia* or leukocythemia* or leukocythaemia* or "childhood ALL" or AML or JMML or JCML or lymphoma* or hodgkin* or nonhodgkin* or burkitt* or nonburkitt* or blastoma* or neuroblastoma* or meningioma* or wilm or wilms or nephroblastoma* or rhabdomyosarcoma* or myosarcoma* or sarcoma* or osteosarcoma* or ewing* or retinoblastoma* or teratoma* or HCC or (hepatocellular NEXT carcinoma*) or (hepatic NEXT carcinoma*) or hepatoblastoma* or hepatoma* or pnet or medulloblastoma* or ganglioma* or glioma* or atrt or pancreatoblastoma* or glioblastoma* or (infantile NEXT fibrosarcoma*) or egct or jgct or ependymoma* or pxa or xanthoastrocytoma* or oligodendroglioma* or astrocytoma* or ("choroid plexus" NEXT papilloma*) or ("choroid plexus" NEXT carcinoma*) or dipg or gliofibroma* or pmmti or dscrct or (synovial NEXT sarcoma*)):ti,ab,kw  #1 or #2 or #3  [mh pediatrics] or [mh pediatricians] or [mh "nurses, pediatric"] or [mh ^"pediatric nurse practitioners"] or [mh ^"pediatric assistants"] or [mh ^"adolescent medicine"] or [mh ^"hospitals, pediatric"] or [mh "pediatric nursing"] or [mh "intensive care units, pediatric"] or [mh ^"intensive care, neonatal"] or [mh infant] or [mh child] or [mh ^adolescent] or [mh ^"child health"] or [mh ^"infant health"] or [mh ^"adolescent health"] or [mh "child health services"] or [mh ^"adolescent health services"]  (pediatric* or paediatric* or infan* or baby* or babies or neonat* or newborn* or new-born* or child* or kid or kids or schoolchild* or (school NEXT age*) or schoolage* or (primary NEXT school*) or (elementary NEXT school*) or (primary NEXT age*) or (elementary NEXT age*) or (secondary NEXT school*) or (high NEXT school*) or highschool* or preschool* or pre-school* or nursery or toddler* or kindergar* or adoles* or teen* or youth or youths or "young people" or (young NEXT person*) or (young NEXT adult*) or preteen* or pre-teen* or pubescen* or prepubescen* or pre-pubescen* or boy* or girl* or nicu or picu or juvenile* or preadolescen*):ti,ab,kw  #5 or #6  [mh ^fatigue] or [mh ^"mental fatigue"] or [mh ^"muscle fatigue"] or [mh ^lethargy] or [mh ^sleepiness] or [mh "physical endurance"] or [mh ^asthenia]  (fatigu* or tired* or tire or weary or weariness or wearied or endurance or exhaustion or exhausted or letharg* or listless* or sleepy or sleepiness or drowsy or drowsiness or somnolen* or astheni* or ((reduced or loss or low or diminished or less) NEAR/2 (energy or vitality)) or lassitude):ti,ab,kw  #8 or #9  #4 and #7 and #10 = 1826  **Grey Literature Search Strategy**  CPG Infobase  cancer AND fatigue    Cancer Guidelines Database  fatigue    ECRI Guidelines Trust  cancer AND fatigue    Canadian Cancer Trials  fatigue    clinicaltrials.gov  fatigue \| Childhood Cancer \| First posted from 01/01/2022 to 12/31/2023    Google (first 10 pages)  (cancer\|neoplasm\|tumor\|oncology) (child\|childhood\|pediatric) (fatigue\|tired\|exhaustion\|endurance\|energy) ext:pdf with date limit Jan 1, 2022 - Today    Total = 155 |
| --- |

**Table S2. JBI’s critical appraisal checklist for assessing the study quality for studies reporting prevalence data**

| **Checklist Questions** | **Options** | **Scoring Criteria** |
| --- | --- | --- |
| Was the sample frame appropriate to address the target population? | Yes, No, Unclear | Appropriate if 80% of eligible population was enrolled or random sampling was used. |
| Were study participants sampled in an appropriate way? | Yes, No, Unclear | Appropriate if random or if all patients were approached. Convenience sampling was considered not appropriate. |
| Was the sample size adequate? | Yes, Unclear | Based on sample size calculation done by the study. No, if no sample size calculation were provided. Unclear if calculation is missing. |
| Were the study subjects and the setting described in detail? | Yes, No, Unclear | Requires description of inclusion criteria, demographics, cancer type, and detailed treatment specifics. |
| Were valid methods used for the identification of the condition? | Yes, No | Yes if a validated fatigue measure/scale was used |
| Was the condition measured in a standard, reliable way for all participants? | Yes, No, Unclear | Considered reliable if all participants received the same fatigue questionnaire in a similar way to report fatigue |
| Was there appropriate statistical analysis? | Yes, No, Not Applicable | For prevalenace outcome, yes prevalence was calculate and reported accurately. |
| Was the response rate adequate? | Yes (≥80%), No (<80%) | Yes if response rate was 80% or more. |

### **Table S3. Summary of fatigue scales employed in the included studies (N = 47)**

| **Type of fatigue scale^a^** | **Number of studies** | **%^b^** |
| --- | --- | --- |
| FS-C, FS-A, FS-P, FS-S | 17 | 36 |
| PedsQL-MFS | 8 | 17 |
| PROMIS | 7 | 15 |
| Memorial Memorial Symptom Assessment Scale | 5 | 11 |
| SSPedi | 3 | 6 |
| PRO-CTCAE | 2 | 4 |
| Likert Scale | 2 | 4 |
| Daily Fatigue Report Form | 1 | 2 |
| Fatigue Scale for Children with Cancer | 1 | 2 |
| Life Situation Scale for Children | 1 | 2 |
| Therapy-Related Symptom Checklist child version | 1 | 2 |
| Visual Analogue Scale | 1 | 2 |
| Symptom assessment scale for patients | 1 | 2 |

^a^18 studies used both parent- and patient-reported versions, 7 studies used only parent-reported versions, 21 studies used only patient-reported versions, and one study did not specify.

^b^Three studies used more than one type of fatigue scale; hence the denominator is 49

Abbreviations: FS-A: Fatigue Scale-Adolescent, FS-C: Fatigue Scale-Child, FS-P: Fatigue Scale-Parent, FS-S: Fatigue Scale-Staff; MSAS:, PedsQL-MFS: Pediatric Quality of Life Multidimensional Fatigue Scale, PRO-CTCAE: Patient-reported outcome Common Terminology Criteria for Adverse Events, PROMIS: The Patient-Reported Outcomes Measurement Information System, SSPedi: Symptom Screening in Pediatrics tool

### **Supplemental Table S4. Prevalence of overall fatigue reported in the included studies (N = 26)**

| **Study ID** | **Country** | **Age of participants in years** | **Type of cancer** | **Type cancer treatment** | **Phase of cancer treatment** | **Study Setting** | **Type of fatigue scale** | **Person reporting fatigue** | **Total number of patients enrolled** | **No of patients assessed for fatigue** | **No of patients with fatigue** | **Percentage of participants with fatigue** |
| --- | --- | --- | --- | --- | --- | --- | --- | --- | --- | --- | --- | --- |
| Gandy et al. 2022^76^ | USA | 8.3 (3 to 16) | CNS tumor | Radiation | Prior to radiotherapy and weekly through the end of radiotherapy | NR | FS-P | Caregiver | 37 | 33 | 30 | 90.9 |
| Irestorm et al. 2022^77^ | Netherlands | 6.3 (NR) | ALL | Chemotherapy | 5 months after diagnosis, 12 months after diagnosis, 24 months after diagnosis, and 12 months after end of treatment. | NR | PedsQL MFS | Parent | 127 | 92 | 72 | 78.3 |
| Jacobs et al. 2022^78^ | USA and Canada | 13.0 (7 to 18) | LL | Chemotherapy | Within 72 h before the start of a chemotherapy treatment cycle | Both | PROMIS | Patient | 257 | 257 | 175 | 68.1 |
| Weaver et al. 2022^79^ | USA | 13.0 (7 to 17) | Mixed | Chemotherapy/radiation | Within 72h of beginning next cycle of treatment;, 7 to 17 days later for patients receiving chemotherapy, and 4 weeks later for patients receiving radiation | Both | PROMIS | Patient | 492 | 436 | 303 | 69.5 |
| Bradford et al. 2021^81^ | Australia | 12 (8 to 18) | Mixed | NR | NR | no | SSPedi | Patient | 48 | 48 | 22 | 45.8 |
| Cheng et al. 2021^82^ | China | NR | Mixed | Chemotherapy/radiation/surgery | NR | NR | PROMIS | Patient | 187 | 187 | 93 | 49.7 |
| Cheng KK et al. 2021^83^ | Singapore | 13.7 (10 to 18) | Mixed | Chemotherapy | Baseline (after diagnosis and before the 1st cycle of chemotherapy); first 2 weeks in each cycle of chemotherapy, and at 6 months after baseline. | yes | MSAS | Patient | 50 | 50 | 32 | 64.0 |
| Li et al. 2020 | China | 8.9 (NR) | ALL and AML | Chemotherapy | Induction/consolidation/maintenance | no | MSAS | Parent and patient | 159 | 159 | 143 | 89.9 |
| Rostagno et al. 2020^87^ | Italy | 11.7 (5 to 17) | Mixed | Chemotherapy | First chemotherapy cycle and after 4, 8, and 12 months. | NR | PedsQL-MFS | Parent and patient | 134 | 134 | 118 | 88.1 |
| Cadamuro et al. 2020^89^ | Brazil | NR | Mixed | Chemotherapy/radiation/surgery | NR | yes | SSPedi | Parent and patient | 157 | 157 | 98 | 62.4 |
| Kudubes et al. 2019^90^ | Turkey | 9.4/9.1 (control/experimental group, NR) | Mixed | Chemotherapy/radiation/surgery | non-specified | NR | Symptom assessment scale for patients | Parent and patient | 80 | 80 | 78 | 97.5 |
| Rogers et al. 2019^92^ | USA | 9.5 (4 to 19) | Medulloblastoma | Autologous HCT | NR | yes | FS-C, FS-A, FS-P | Parent and patient | 43 | 33 | 20 | 60.6 |
| Macpherson et al. 2018^40^ | USA | (8 to 18) | Mixed | Chemotherapy | Beginning of chemotherapy cycle | Both | FS-C, FS-A, PROMIS | Patient | 96 | 96 | 45 | 46.9 |
| Bastani et al. 2015^57^ | Iran | 10.0 (8 to 12) | ALL | Chemotherapy | Induction | Inpatient | VAS | Patient | 120 | 120 | 117 | 97.5 |
| MDR Nunes et al. 2015^97^ | USA | 12.8 (8 to 17) | Mixed | NR | NR | Outpatient | PedsQL-MFS | Patient | 42 | 35 | 19 | 54.3 |
| Ameringer et al. 2013^99^ | USA | 15.3 (13 to 18) | Mixed | NR | NR | Both | FS-A | Patient | 9 | 9 | 9 | 100.0 |
| Hinds et al. 2013^43^ | USA | 12.9 (8 to 17) | Mixed | NR | NR | Both | PROMIS | Patient | 93 | 90 | 60 | 66.7 |
| Miller et al. 2011^102^ | USA | 13.5 (10 to 17) | Mixed | NR | Day 1 of hospitalization | Inpatient | MSAS | Patient | 39 | 39 | 24 | 61.5 |
| Baggott et al. 2010^3^ | USA | 14.8 (10 to 18) | Mixed | Chemotherapy | NR | Both | MSAS | Patient | 66 | 56 | 43 | 76.8 |
| Dupuis et al. 2010^103^ | Canada | 9.4 (4 to 18) | Mixed | NR | NR | Both | Likert scale | Parent | 200 | 158 | 126 | 79.7 |
| Erickson et al. 2010^18^ | USA | 16.1 (12 to 19) | Mixed | Chemotherapy | NR | NR | Daily Report form | Patient | 25 | 20 | 20 | 100 |
| Walker et al. 2010^17^ | USA | 14.2 (10-19) | Mixed | Chemotherapy/radiation/surgery | One week before chemotherapy | Both | MSAS | Patient | 51 | 46 | 28 | 60.9 |
| Sitaresmi et al. 2009^106^ | Indonesia | 7.1 (2 to 16) | ALL | Chemotherapy | Induction/maintenance | Both | Likert scale | Parent | 51 | 51 | 29 | 56.9 |
| Yeh et al. 2009 | Taiwan | 14.2 (10 to 18.9) | Mixed | NR | NR | Both | PedsQL-MFS | Patient | 108 | 108 | 57 | 52.8 |
| Enskar et al. 2008^109^ | Sweden | 9.6 (NR) | Mixed | NR | NR | Both | LSS-C | Patient | 17 | 17 | 11 | 64.7 |
| Williams et al. 2006^113^ | USA | 10.4 (2 to 18) | Mixed | Chemotherapy | NR | Inpatient | TRSC | Patient | 11 | 11 | 10 | 90.9 |

###

### Abbreviations: FS-A: Fatigue Scale-Adolescent, FS-C: Fatigue Scale-Child, FS-P: Fatigue Scale-Parent, FS-S: Fatigue Scale-Staff, HCT: Hematopoietic stem cell transplant, LSS-C: Life Situation Scale for Children, MSAS: Memorial Symptom Assessment Scale, NR- not-reported, PedsQL-MFS: Pediatric Quality of Life Multidimensional Fatigue Scale, PROMIS: The Patient-Reported Outcomes Measurement Information System, SSPedi: Symptom Screening in Pediatrics tool, TRSC: Therapy-Related Symptom Checklist child version, USA: United States of America, VAS: Visual Analog Scale

### **Table S5. Prevalence of severe fatigue reported in individual study (N = 8)**

| **Study ID** | **Country** | **Age of participants in years** | **Type of cancer** | **Type cancer treatment** | **Phase of cancer treatment** | **Study Setting** | **Type of fatigue scale** | **Person reporting fatigue** | **Total number of patients enrolled** | **No. of patients assessed for severe fatigue** | **No. of patients with severe fatigue** | **Percentage of participants with severe fatigue** | **Cut off score for the severe fatigue** |
| --- | --- | --- | --- | --- | --- | --- | --- | --- | --- | --- | --- | --- | --- |
| Gandy et al. 2022^76^ | USA | 8.3 (3 to 16) | CNS tumor | Radiation | Prior to radiotherapy and weekly through the end of radiotherapy | NR | FS-P | Caregiver | 37 | 33 | 29 | 87.9 | 41 or higher |
| Rostagno et al. 2020^87^ | Italy | 11.7 (5 to 17) | Mixed | Chemotherapy | First chemotherapy cycle and after 4, 8, and 12 months | NR | PedsQL-MFS | Parent and patient | 134 | 93 | 2 | 2.1 | 7 or higher |
| Tomlinson et al. 2019^115^ | Canada and USA | NR | Mixed | NR | NR | yes | SSPedi | Patient | 366 | 366 | 108 | 29.5 | score of 3  or 4 |
| Bastani et al. 2015^57^ | Iran | 10.0 (8 to 12) | ALL | Chemotherapy | Induction | Inpatient | VAS | Patient | 120 | 120 | 16 | 13.3 | score 7 to 10 on VAS |
| Dupuis et al. 2010^103^ | Canada | 9.4 (4 to 18) | Mixed | NR | NR | Both | Likert scale | Parent | 200 | 158 | 85 | 53.8^a^ | NR |
| Sitaresmi et al. 2009^106^ | Indonesia | 7.1 (2 to 16) | ALL | Chemotherapy | Induction/maintenance | Both | Likert scale | Parent | 51 | 51 | 8 | 15.7 | 4 or more on the Likert scale |
| Yeh et al. 2008^111^ | Taiwan | 14.2 (10 to 18.9) | Mixed | NR | NR | Both | PedsQL-MFS | Patient | 108 | 108 | 30 | 20.8 | NR |
| Williams et al. 2006^113^ | USA | 10.4 (2 to 18) | Mixed | Chemotherapy | NR | Inpatient | TRSC | Patient | 11 | 11 | 2 | 18.2 | score of 3 or 4 |

Abbreviations: FS-P: ALL; Acute Lymphoblastic Leukemia; CNS; Central Nervous System: Fatigue Scale-Parent, NR- not-reported, PedsQL-MFS: Pediatric Quality of Life Multidimensional Fatigue Scale, SSPedi: Symptom Screening in Pediatrics tool, TRSC: Therapy-Related Symptom Checklist child version, USA: United States of America, VAS: Visual Analog Scale

^a^Moderately to extremely severe fatigue

### **Figure S1. Study quality assessment of all studies included in the systematic review (N=47)**

### **Figure S2. Study quality assessment of studies contributing to the primary outcome of overall fatigue prevalence**

###


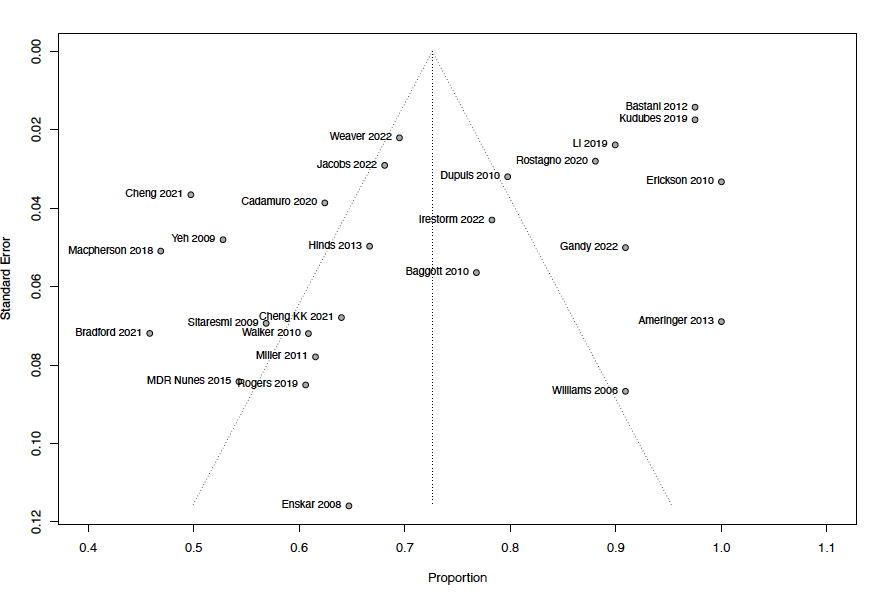


**Figure S3. Funnel plot assessing the publication bias**
